# Perio-dontal - Neuro-immune axis: Expression analysis of Vitamin D and brain-derived neurotrophic factor in periodontitis patients with neuro-psychiatric disorders

**DOI:** 10.6026/973206300211016

**Published:** 2025-05-31

**Authors:** Jaideep Mahendra, Devadharshini Chandrasekar, Krithika C., Baiju R.M, Muskan Bedi, Karunanidhi K., Yasin Begam

**Affiliations:** 1 Department of Periodontics, Meenakshi Ammal Dental College and Hospital, Chennai, Tamil Nadu, India; 2 Department of Oral Medicine, Meenakshi Academy of Higher Education and Research, Chennai, Tamil Nadu, India; 3 Department of Periodontics, Government Dental College, Kottayam, Kerala, India; 4 Department of General Medicine, Sri Ramachandra Medical College and Research Institute, Chennai, Tamil Nadu, India; 5 Department of Research, Meenakshi Academy of Higher Education and Research, Chennai, Tamil Nadu, India; 6 Department of Biochemistry, Meenakshi Ammal Dental College and Hospital, Chennai, Tamil Nadu, India

**Keywords:** Periodontitis, neuropsychiatric disorders, vitamin D, brain-derived neurotrophic factor (BDNF), salivary biomarkers, mental health

## Abstract

The association between periodontal health, neuropsychiatric status and metabolic indicators by assessing salivary Vitamin D and brain-derived neurotrophic factor (BDNF) levels is of interest. Eighty participants were categorized into four groups based on periodontal and neuropsychiatric status. Patients with both periodontitis and neuropsychiatric disorders showed significantly higher age, BMI, plaque index and probing depth and lower Vitamin D, BDNF levels and mental health scores (p < 0.001). No significant correlation was observed between biochemical markers and periodontal parameters. The findings suggest a potential link between periodontal inflammation and neuropsychiatric conditions, emphasizing the relevance of multidisciplinary care.

## Background:

Periodontitis is a chronic inflammatory disease that causes irreversible destruction of the tooth-supporting structures, often resulting in tooth loss [1]. Recent research has deepened our understanding of periodontitis, revealing it as a source of systemic inflammation linked to various health conditions, such as cardiovascular diseases, diabetes and metabolic syndrome [2]. The complex relationship between periodontitis and neuropsychiatric disorders has become a significant focus of research [3, 4]. Emerging evidence suggests that periodontal disease may not only aggravate but also potentially contribute to neuropsychiatric conditions, emphasising the importance of shared inflammatory mechanisms and overlapping risk factors such as stress and impaired metabolic control [5]. Periodontitis, marked by extensive and severe destruction of periodontal tissues, poses a considerable challenge to both oral and systemic health [6]. Its chronic nature and tendency to increase systemic inflammatory markers may pose a threat to the mental health which makes it a pivotal area of study for understanding the links between oral health and psychosis [7]. A significant factor connecting periodontitis with broader health ailments is Vitamin D, a secosteroid hormone essential for maintaining calcium balance and bone health [8]. Vitamin D regulates immune responses; its deficiency has been linked to the development of both periodontal disease and neuropsychiatric disorders [7]. Evidence from previous literature has shown that individuals with periodontitis, who exhibited Vitamin D deficiency, were associated with heightened infection risk, weakened immune response and chronic inflammation [9]. On the other hand, vitamin D receptors are also present in brain where they play a key role in neurodevelopment and neuroprotection. The presence of Vitamin D receptors in the brain suggests that Vitamin D could be an important marker in progression of periodontitis thereby affecting mental well-being [10].

Brain-derived neurotrophic factor (BDNF) is an essential neurotrophin that supports the survival, development and plasticity of neurons playing a critical role in cognitive functions such as learning and memory [11]. Chronic inflammation, commonly seen in periodontitis, can lead to alterations in BDNF expression disrupting the normal function [12]. This dysregulation leads to reduced levels of this neurotrophin which have been associated with various neuropsychiatric disorders, including depression, anxiety and cognitive decline [13, 14]. Investigating the impact of periodontitis on Vitamin D and Brain-Derived Neurotrophic Factor (BDNF) expression in patients with neuropsychiatric disorders could yield valuable insights into how the oral and systemic inflammation influences the development and progression of neuropsychiatric disorders. This study addresses an under-researched area by investigating the roles of Vitamin D and Brain-Derived Neurotrophic Factor (BDNF) in periodontitis associated neuropsychiatric conditions. Therefore, it is of interest to assess and compare the demographic variables, periodontal parameters and the significant biomarkers of neuropsychiatric disorders such as Vitamin D and BDNF among the groups.

## Materials and Methods:

## Patient selection:

This case-control, cross-sectional study was conducted from December 2023 to June 2024 in Chennai, Tamil Nadu, India and adhered to the ethical guidelines set forth by the Helsinki Declaration as revised in 2013, ensuring that all ethical considerations were addressed. Approval for the study was granted by the institutional ethical committee of Meenakshi Academy of Higher Education and Research, Chennai MADC/IEC/III/84/2023, ensuring compliance with all relevant ethical standards. In total 115 patients were recruited of which 35 patients were excluded since they did not meet the inclusion criteria. Finally, 80 patients were selected based on the inclusion and exclusion criteria.

## Inclusion criteria:

[1] Willingness to participate in the study;

[2] Age between 35-65 years;

[3] Having at least 20 natural teeth;

[4] A clinically healthy periodontium, defined as Modified Gingival Index (MGI) scores of less than 1 and no clinical attachment loss (CAL=0) (for Group H & NPD);

[5] For periodontitis (stages II-IV), clinical signs of gingival inflammation, interproximal attachment loss affecting ≥30% of teeth, CAL of ≥3 mm and probing pocket depth (PPD) of ≥4 mm, consistent with the 2017 classification criteria (for group P & P+NPD)

[6] Assessment of neuropsychiatric disorders based on the General Health Questionnaire - 28 (GHQ-28) and confirmed through diagnoses by a certified psychiatrist (for group NPD & P+NPD). Diabetic status was also included to assess the potential impact of diabetes on the study outcomes.

## Exclusion criteria:

[1] History of antibiotic use or periodontal therapy within the past six months;

[2] Current orthodontic therapy;

[3] Pregnancy;

[4] Use of systemic steroids

[5] Presence of any other systemic conditions which could alter the results.

Participants were carefully recruited based on these criteria to ensure a balanced and comparable study population thereby maintaining the integrity and reliability of the study outcomes. Based on the above criteria, participants were grouped as: Group -H included systemically and periodontally healthy participants who were selected from the general hospital of Meenakshi Ammal General Hospital, while the volunteers came for master health check-up and Group - P comprised of periodontitis patients with good systemic health who were selected from the Department of Periodontology at Meenakshi Ammal Dental College and Hospital. Group -NPD consisted of neuropsychiatric patients with good periodontal health. Group- P+NPD involved neuropsychiatric patients with periodontitis. NPD and P+NPD were selected from neurology and psychiatric wards of various tertiary medical centres in Chennai and surrounding areas. All participants provided written informed consent prior to participate in the study (Figure 1 see PDF).

## Sample size:

The sample size for the study was determined based on the prevalence of periodontal disease and neuropsychiatric disorders in the region, as observed from hospital records and supported by findings from prior literature. A power analysis was performed to ensure statistical reliability and it was calculated that including a minimum of 20 participants per group would provide 90% statistical power. This approach ensured that the sample size was adequate to detect meaningful differences across the study groups while maintaining scientific rigor.

## Parameters assessed:

On the day of assessment, key demographic, periodontal, psychological parameters were systematically recorded from the data extracted from the medical records of patients. The demographic information collected included age and Body Mass Index (BMI) which were recorded to evaluate potential correlations between these factors and clinical outcomes in the context of periodontal and neuropsychiatric health. Periodontal health was evaluated using standardized clinical indices to ensure accurate and reproducible results. The Plaque Index (Silness and Loe) was employed to assess plaque accumulation and the Oral Hygiene Index - Simplified (OHI-S) by Greene and Vermillion was used to determine the overall oral hygiene status of participants. Probing Pocket Depth (PPD) and Clinical Attachment Level (CAL) were measured using a Williams Periodontal Probe* (Hu-Friedy, Chicago, IL, USA) by two trained examiners (J.M and D.C.). Calibration of examiners was conducted on a sample of 10 patients independent of the study population, with Kappa statistics calculated to assess inter- and intra-examiner reliability, yielding satisfactory scores of 0.73 and 0.71, respectively. The PPD and CAL were recorded at six different sites per tooth (mesiobuccal, buccal, distobuccal, mesiolingual, lingual and distolingual), providing a detailed assessment of periodontal disease severity. The psychological status of participants was assessed using the General Health Questionnaire - 28 (GHQ-28), a standardized tool that evaluates mental health across four domains: somatic symptoms, anxiety and insomnia, social dysfunction and depression. Participants were asked to reflect on their general health over the past few weeks and respond to behavioural items using a 4-point scale: "not at all," "no more than usual," "rather more than usual" and "much more than usual." The scoring followed the original Likert scale (0, 1, 2, 3), with total scores ranging from 0 to 84. Higher scores indicated greater psychological distress. According to established guidelines, participants with scores ≤23 were classified as non-psychiatric, while those scoring ≥24 were categorized as psychiatric. This evaluation facilitated an in-depth understanding of the interplay between psychological health, oral health and biochemical markers in the study population [15].

## Unstimulated saliva sample collection:

Unstimulated 5 ml whole saliva samples were collected from the patients two hours after their last meal. All participants were instructed to refrain from eating or drinking for one hour prior to saliva collection. Each participant was asked to rinse their mouth thoroughly with distilled water before expectorating unstimulated whole saliva into a 50 ml centrifuge tube, with a final saliva volume of 3 to 5 ml obtained. The collected saliva samples were transported to the laboratory within 24 hours, using a standard gel coolant pack to maintain a temperature between 2°C and 4°C.

## Molecular analysis:

Measurement of Vitamin D, BDNF and Random Blood Glucose levels were completed. The expression levels of salivary Vitamin D and Brain-Derived Neurotrophic Factor (BDNF) were quantified using enzyme-linked immunosorbent assay (ELISA) kits from Bioassay Technology Laboratory (Catalogue No: E1302Hu for Vitamin D and Catalogue No: E1543Hu for BDNF). Saliva samples were first warmed to room temperature and centrifuged to remove particulate matter, with the supernatant subsequently diluted as per the manufacturer's recommendations. For Vitamin D analysis, the diluted samples, along with standards and controls, were added to pre-coated microtiter plates and incubated with detection antibodies. After washing, a substrate solution was introduced and the resultant colour intensity, proportional to the Vitamin D concentration, was measured using a spectrophotometer. Similarly, BDNF analysis involved adding diluted samples to microtiter plates specific to BDNF, incubating with a biotinylated detection antibody and following with a wash step. A streptavidin-enzyme conjugate was then introduced and a colour reaction was developed, with the intensity measured spectrophotometrically. For both assays, concentrations were calculated by comparing optical density readings to standard curves generated with known concentrations of Vitamin D or BDNF. All procedures adhered strictly to the manufacturer's protocols to ensure accuracy and processed saliva samples were safely incinerated post-analysis following standard laboratory protocols. The estimation of Random Blood Glucose (RBG) levels was incorporated into the study by retrieving data from hospital records. This parameter was included to evaluate the metabolic status of participants and its potential influence on periodontal and systemic health. The recorded RBG levels provided a comprehensive understanding of the participant's glycaemic control, offering valuable insights into the interplay between glucose metabolism, periodontal parameters and neuropsychiatric conditions.

## Statistical analysis:

All statistical analyses were performed using SPSS version 17 for Windows. The statistical analysis incorporated both parametric and non-parametric tests to compare differences across the four study groups. Descriptive statistics, such as mean and standard deviation, were used to summarize the data for each variable. To examine differences between groups, the One-Way Analysis of Variance (ANOVA) was applied, for identifying statistically significant differences in continuous variables (*e.g.*, age, BMI, plaque index and RBG levels) when comparing multiple groups. For a significant p-value (p < 0.05), post hoc tests were performed to determine specific group differences. Additionally, Pearson's correlation test, a parametric method, was used to correlate the biochemical parameters (Vitamin D, BDNF, RBG) and periodontal variables (*e.g.*, plaque index, OHI-S, CAL, PPD). This test was chosen to assess the strength and direction of correlations among continuous variables that met normality assumptions. The threshold for statistical significance was set at p < 0.05 for all analyses. These comprehensive statistics enabled a thorough assessment of the associations and significant differences in demographic, biochemical and clinical variables, enhancing insights into the relationships between periodontal health and neuropsychiatric conditions across the study groups.

## Results:

The analysis of demographic variables across the four study groups revealed that the participants in P + NPD group were significantly older (55.40 ± 13.648) than those in the other three groups (p < 0.001). Body Mass Index (BMI) also differed between the groups, with the highest mean observed in the P group (24.520 ± 3.3673) with a statistically significant difference (p = 0.04). Periodontal assessments indicated that the participants in P+NPD group had significantly higher mean scores in Plaque Index (PI) (2.431 ± 0.40) and Oral Hygiene Index-Simplified (OHI-S) (3.10 ± 0.629) than the other groups (p < 0.001). Furthermore, probing pocket depth (PPD) and clinical attachment level (CAL) were also significantly elevated in this group, with the mean of 6.07±0.84 and 5±0.951 respectively, indicating more severe periodontal attachment loss (p < 0.001) (Table 1). Biochemical markers showed notable differences among the groups (Figure 2 see PDF). Vitamin D levels were lowest in P+NPD group with the mean of 0.33 ± 0.114. Brain-Derived Neurotrophic Factor (BDNF) levels were lowest in P+NPD group with the mean of 0.311±0.055, which was significant compared to other groups (p = 0.01). Random Blood Glucose (RBG) levels were significantly higher in participants in P+NPD group with a higher mean of 179.077±47.26 (p < 0.001) (Table 2). Psychological assessment scores on the GHQ-28 scale showed a significant increase in participants in P+NPD group with the mean of 48.13 ± 10.50 compared to the other groups highlighting a pronounced psychological disparity in the P+NPD group (p < 0.001) (Table 3). On undergoing Correlation Analysis between the biochemical markers (Vitamin D, BDNF, RBG) and clinical periodontal parameters (plaque score, OHI-S, CAL, PPD) across the groups, the parameters did not positively correlate indicating that these biochemical markers may not directly correlate with periodontal status among the study participants (Table 4.

## Discussion:

The current study provides novel insights exploring the role of Vitamin D and Brain-Derived Neurotrophic Factor (BDNF) in patients with periodontitis and neuropsychiatric disorders, highlighting the complex interplay between periodontal inflammation, metabolic dysregulation and mental health. We hypothesise that both Vitamin D deficiency and altered BDNF expression may serve as key biomarkers linking periodontal disease to neuropsychiatric conditions, adding to the growing body of evidence on the systemic impact of periodontitis. The demographic variables of the study participants particularly age and body mass index (BMI), demonstrated statistically significant differences across the four study groups and providing insight into the interplay between age, BMI and periodontal status with neuropsychiatric disorders. Age emerged as a significant factor, with individuals presenting with both periodontitis and neuropsychiatric disorders being notably older compared to those in the other groups. This finding aligns with existing literature that suggests advancing age as a shared risk factor for both periodontal disease and neuropsychiatric conditions. Huang *et al.* observed that periodontal disease is more prevalent among middle-aged and older adults compared to younger individuals. These age groups tend to exhibit reduced awareness and prioritization of periodontal health, potentially contributing to the higher incidence of periodontal issues [16]. Similarly, age has been linked to morphological changes in the brain, which often contribute to the onset of neuropsychiatric disorders, making many elderly individuals more susceptible to these conditions [17, 18]. Busse *et al.* investigated the seroprevalence of N-methyl-D-aspartate receptor (NMDA-R) autoantibodies in aging individuals without neuropsychiatric disorders and in dementia patients. His findings suggest that the presence of these autoantibodies increases with age, particularly in dementia patients, indicating a potential age-related susceptibility to neuropsychiatric conditions linked to autoimmune mechanisms [19]. Aich *et al.* highlighted that the prevalence of neuropsychiatric disorders in older adults appears consistent across developed and developing nations [20]. The heightened susceptibility of older adults to inflammatory and metabolic dysregulation could provide a plausible explanation for this observation. BMI has long been recognized as a key indicator of overall health and its association with both periodontal disease and neuropsychiatric disorders highlights the complex interplay between metabolic dysregulation and mental health [21]. Elevated BMI is often associated with systemic inflammation, which could exacerbate both periodontal disease and neuropsychiatric conditions, supporting the notion that metabolic factors are crucial in understanding the systemic impact of periodontitis [22]. This finding emphasizes the need for a multidisciplinary approach to managing patients with periodontal and neuropsychiatric disorders, considering both metabolic and inflammatory factors as contributors to disease progression. Herrera *et al.* conducted a systematic review and meta-analysis, confirming a strong association between obesity and periodontitis [23]. Their findings highlighted an increased risk of periodontitis among overweight and obese individuals, suggesting that elevated BMI contributes to periodontal disease development. This aligns with the current study, where BMI differences across groups showed a clear connection between metabolic factors and periodontal health. Kolte *et al.* concluded that their study found a positive and strong correlation between anxiety, obesity and periodontal disease. This finding reinforces the complex interrelationship between mental health, metabolic dysregulation and oral health [24]. The analysis of periodontal parameters showed significant differences among the study groups. Plaque and OHI-S scores were highest in participants in P+NPD group (p < 0.001), reflecting challenges in maintaining oral hygiene. Similarly, greater periodontal pocket depth (PPD) and clinical attachment level (CAL) were observed in this group, indicating advanced periodontal destruction. This was in accordance with prior studies, which have linked elevated inflammatory markers in periodontitis to neuropsychiatric outcomes [25, 26]. Recent research by Shen *et al.* demonstrated that restoration of periodontal tissue homeostasis mitigated cognitive decline by reducing Serpina3nhigh astrocytes in the hippocampus, highlighting a critical link between periodontal health and neurocognitive function [27]. In light of the findings from the systematic review by Said-Sadier *et al.* it is evident that chronic periodontitis is significantly associated with an increased risk of cognitive decline and dementia, particularly among individuals with prolonged exposure to periodontal disease [28]. These findings highlight the compounded impact of neuropsychiatric conditions on periodontal health. Czyz and Firkova in their studies mentioned that the chronic inflammation associated with periodontitis has long been recognized as a driver of systemic diseases, including cardiovascular and metabolic disorders [29]. The current study extends these findings by demonstrating that systemic inflammation, as indicated by periodontal parameters, is also associated with disruptions in neurotrophic signalling, particularly via BDNF. This may also be associated with the fact that neuropsychiatric patients often have difficulty maintaining proper oral hygiene due to cognitive impairments and motor dysfunction [30]. This was in accordance with the study conducted by Kilijanska *et al.* who reported that anxiety negatively influences oral health status, with higher levels of dental anxiety being associated with poorer dental and periodontal health [31]. The biochemical analysis across the four study groups revealed significant variations, shedding light on the systemic implications of periodontitis and neuropsychiatric disorders. Random blood glucose (RBG) levels were highest in participants P+NPD group, significantly exceeding those of other groups (p < 0.001). This was in accordance with Graziani *et al.* who in his systematic review and meta-analysis, highlights that periodontitis significantly affects diabetes control, its incidence and the associated complications [32]. On the other hand Lee *et al.* found a significant association between psychiatric disorders and an increased risk of developing type 2 diabetes (T2D), particularly highlighting that young adults with schizophrenia and bipolar disorder are at a higher risk [33]. This suggests a potential link between periodontal inflammation and metabolic health in patients with neuropsychiatric disorder.

Vitamin D levels were found to be lower in participants in P+NPD group (p < 0.001), underscoring its potential role in the pathogenesis of both conditions. Previous studies have demonstrated that Vitamin D deficiency has been associated with impaired immune modulation, heightened inflammation and compromised neuronal function, aligning with the observed findings [34, 35]. Evidence from a recent randomized double-blind placebo-controlled trial by Peric *et al.* suggests that a six-month regimen of Vitamin D supplementation significantly enhances the outcomes of non-surgical periodontal therapy in patients with Vitamin D deficiency, highlighting the vitamin's critical role in modulating periodontal inflammation and supporting tissue healing [34]. Recently Jamalian *et al.* in their systematic review and meta-analysis highlighted that vitamin D supplementation may positively influence mental health outcomes in individuals with psychiatric disorders by modulating inflammatory and oxidative stress biomarkers [35]. These finding supports the association of vitamin D levels with both periodontal and neuropsychiatric health. These findings corroborate earlier research linking low Vitamin D levels to both chronic periodontitis and mental health issues, particularly depression and cognitive dysfunction [36]. The immunomodulatory effects of Vitamin D have shown to be critical in mitigating the inflammatory response in both periodontitis and neuropsychiatric conditions [37]. Our findings highlight the critical role of Vitamin D in modulating inflammation and neuronal function in individuals with coexisting oral and mental health disorders. Given the established presence of Vitamin D receptors in the brain and their involvement in neuroprotection, these results align with the hypothesis that Vitamin D is a key regulator in the interplay between periodontal and neuropsychiatric health [38]. The present study adds to the emerging evidence on the role of BDNF in neuropsychiatric disorders and periodontal diseases [39]. Our findings revealed that BDNF levels were significantly reduced in participants with neuropsychiatric disorders, regardless of periodontal health (p = 0.01). In line with this, a study by Teng *et al.* found that decreased BDNF levels were associated with depression and cognitive decline, further suggesting the role of BDNF in mental health disorders [40]. Similarly, a study by Correa *et al.* reported a significant reduction in BDNF levels in individuals with periodontal disease, supporting the notion that BDNF may play a key role in the pathophysiology of both neuropsychiatric and periodontal conditions [14]. Lower levels of BDNF in patients with these conditions suggest that chronic inflammation may inhibit the expression of BDNF [41]. Findings from Xu *et al.* suggest that the administration of Brain-Derived Neurotrophic Factor (BDNF) exerts significant anti-inflammatory and anti-apoptotic effects in the context of bacterial infections, indicating its potential role in mitigating inflammatory responses and neuronal damage, which may have implications for understanding the pathways linking periodontal disease and neuropsychiatric disorders [42]. Chen *et al.* in their investigation revealed that Brain-Derived Neurotrophic Factor (BDNF) exhibits downregulated expression and hypermethylation in periodontitis tissues, suggesting its potential as a biomarker and therapeutic target, thereby reinforcing the notion that alterations in BDNF may significantly influence the pathophysiology of periodontal disease and its associated neuropsychiatric implications [43]. BDNF is essential for neuronal survival and plasticity and its dysregulation has been linked to cognitive impairment and mood disorders [44]. This supports the hypothesis that periodontal inflammation may exacerbate systemic inflammatory pathways, thereby influencing brain health [45]. The dual impact of inflammatory mediators on both periodontal tissues and the central nervous system suggests that improving periodontal health could potentially ameliorate neuropsychiatric symptoms [46]. These findings highlight the complex biochemical interplay between periodontal and neuropsychiatric health, emphasizing the need for a multidisciplinary approach to managing these coexisting conditions. Further studies are warranted to explore the mechanistic links and potential therapeutic interventions targeting these biochemical pathways.

The comparison of GHQ-28 scores across the four study groups revealed significant differences, highlighting the mental health impact of periodontitis and neuropsychiatric disorders. Participants with P+NPD group exhibited the highest GHQ-28 scores (48.13 ± 10.51), indicating a more severe psychological burden compared to the other groups (p < 0.001). This suggests that the co-occurrence of periodontal disease and neuropsychiatric conditions significantly exacerbates mental health symptoms. Consequently, participants in Group P had a moderate GHQ-28 score (18.13 ± 2.67), while those participants in NPD group had notably lower (30.47 ± 3.62). This is in accordance with the previous literature where Babicki *et al.* revealed that frontline medical workers, particularly those who were involuntarily assigned to care for COVID-19 patients, exhibited significantly higher GHQ-28 scores, indicating a greater level of mental distress [47]. These findings support the hypothesis that periodontal disease may contribute to increased psychological distress, particularly in those already affected by neuropsychiatric conditions. The use of the GHQ-28 is significant in this context as it provides a comprehensive assessment of mental health, specifically measuring psychological distress and identifying potential psychiatric disorders [48]. This study offers valuable insights into the intricate connection between periodontal disease, neuropsychiatric disorders and overall systemic health. Notably, it is the first study to apply the GHQ-28 tool in periodontitis patients with neuropsychiatric disorders, providing a novel perspective on the mental health challenges faced by individuals dealing with both periodontal inflammation and neuropsychiatric conditions. The study also emphasizes the critical role of Vitamin D and BDNF in modulating oral and systemic inflammation with mental health, suggesting that interventions targeting these factors may enhance both periodontal and psychological outcomes. Future research should investigate whether periodontal therapies that regulate inflammation can offer additional benefits for mental health via BDNF regulation. Moreover, exploring the potential of salivary biomarkers such as Vitamin D and BDNF as potential markers for both periodontal and neuropsychiatric conditions could enable earlier identification of these disorders and more effective, personalized interventions. However, this study has certain limitations that should be considered. The cross-sectional design limits the ability to draw causal conclusions regarding the relationship between periodontal disease and neuropsychiatric disorders. Additionally, the study's sample size may not fully capture the variability in these conditions across different populations and further research with larger and more diverse cohorts is needed to validate the findings. The biochemical analysis focused on a limited set of biomarkers. The future studies can include other potential inflammatory mediators and neurotrophic factors that may further show significant connection between oral and mental health. Despite these limitations, this study lays the groundwork for future research into the bi-directional relationship between periodontal and neuropsychiatric health and underscores the importance of considering periodontal health and good oral hygiene for mental well-being and vice-versa.

## Conclusion:

A significant correlation between salivary levels of Vitamin D and Brain-Derived Neurotrophic Factor (BDNF) among patients with periodontitis and neuropsychiatric disorders is shown. These biomarkers show potential as non-invasive diagnostic indicators for early detection and integrated management of comorbid conditions. The findings provide a basis for future investigations into the periodontal-neuroimmune axis and personalized therapeutic approaches.

## Figures and Tables

**Table 1:** Comparison of demographic variables and periodontal parameters between four study groups

		**Mean**	**Std. deviation**	**p-value**
Plaque	Systemically Healthy and periodontally healthy subjects	1.6	0.4	<0.001*
	Systemically healthy subjects with Generalised periodontitis	2.266	0.21	
	Periodontally healthy subjects diagnosed with neuropsychiatric disorders	1.513	0.43	
	Generalised periodontitis subjects diagnosed with neuropsychiatric disorders	2.431	0.4	
OHI-S	Systemically Healthy and periodontally healthy subjects	2.24	0.38	<0.001*
	Systemically healthy subjects with Generalised periodontitis	2.76	0.24	
	Periodontally healthy subjects diagnosed with neuropsychiatric disorders	2.22	0.56	
	Generalised periodontitis subjects diagnosed with neuropsychiatric disorders	3.1	0.629	
PPD	Systemically Healthy and periodontally healthy subjects	3	0.286	<0.001*
	Systemically healthy subjects with Generalised periodontitis	4.39	0.58	
	Periodontally healthy subjects diagnosed with neuropsychiatric disorders	2.85	0.51	
	Generalised periodontitis subjects diagnosed with neuropsychiatric disorders	6.07	0.84	
CAL	Systemically Healthy and periodontally healthy subjects	3.2	0.34	<0.001*
	Systemically healthy subjects with Generalised periodontitis	5.07	0.64	
	Periodontally healthy subjects diagnosed with neuropsychiatric disorders	3.04	0.87	
	Generalised periodontitis subjects diagnosed with neuropsychiatric disorders	5	0.951	
One Way ANOVA: *p-value<0.05-statistically significant

**Table 2:** Comparison of biochemical parameters between the four study groups

		**Mean**	**Standard deviation**	**p-value**
RBG	Systemically Healthy and periodontally healthy subjects	119.813	9.71	<0.001*
	Systemically healthy subjects with Generalised periodontitis	154.293	27.91	
	Periodontally healthy subjects diagnosed with neuropsychiatric disorders	152.733	45.239	
	Generalised periodontitis subjects diagnosed with neuropsychiatric disorders	179.077	47.26	
Vit-D	Systemically Healthy and periodontally healthy subjects	0.475	0.067	<0.001*
	Systemically healthy subjects with Generalised periodontitis	0.407	0.088	
	Periodontally healthy subjects diagnosed with neuropsychiatric disorders	0.423	0.09	
	Generalised periodontitis subjects diagnosed with neuropsychiatric disorders	0.331	0.114	
BDNF	Systemically Healthy and periodontally healthy subjects	0.462	0.063	0.01*
	Systemically healthy subjects with Generalised periodontitis	0.42	0.07	
	Periodontally healthy subjects diagnosed with neuropsychiatric disorders	0.308	0.037	
	Generalised periodontitis subjects diagnosed with neuropsychiatric disorders	0.311	0.055	
One Way ANOVA: *p-value<0.05-statistically significant

**Table 3:** Comparison of GHQ-28 between four study groups

		**Mean**	**Standard deviation**	**p-value**
	Systemically Healthy and periodontally healthy subjects	16.47	2.326	<0.001*
GHQ-28	Systemically healthy subjects with Generalised periodontitis	18.13	2.669	
	Periodontally healthy subjects diagnosed with neuropsychiatric disorders	30.47	3.623	
	Generalised periodontitis subjects diagnosed with neuropsychiatric disorders	48.13	10.508	
One Way ANOVA: *p-value<0.05-statistically significant

**Table 4:** Correlation between biochemical parameters and periodontal parameters

		**BDNF**	**RBG**	**Plaque**	**OHI-S**	**CAL**	**PPD**
Vit-D	Pearson Correlation	-0.162	0.015	-0.09	0.02	0.098	0.092
	Sig. (2-tailed)	0.215	0.911	0.49	0.87	0.458	0.486
BDNF	Pearson Correlation		-0.215	0.06	0.09	-0.091	-0.038
	Sig. (2-tailed)		0.104	0.61	0.49	0.488	0.776
RBG	Pearson Correlation			0.21	0.25	0.252	0.253
	Sig. (2-tailed)			0.1	0.05	0.056	0.056
-Pearson correlation test
